# Towards self-describing and FAIR bulk formats for biomedical data

**DOI:** 10.1371/journal.pcbi.1010944

**Published:** 2023-03-13

**Authors:** Michael Lukowski, Andrew Prokhorenkov, Robert L. Grossman

**Affiliations:** 1 Center for Translational Data Science, University of Chicago, Chicago, Illinois, United States of America; 2 Section of Biomedical Data Science, Department of Medicine, University of Chicago, Chicago, Illinois, United States of America; 3 Department of Computer Science, University of Chicago, Chicago, Illinois, United States of America; University of Connecticut School of Medicine, UNITED STATES

## Abstract

We introduce a self-describing serialized format for bulk biomedical data called the Portable Format for Biomedical (PFB) data. The Portable Format for Biomedical data is based upon Avro and encapsulates a data model, a data dictionary, the data itself, and pointers to third party controlled vocabularies. In general, each data element in the data dictionary is associated with a third party controlled vocabulary to make it easier for applications to harmonize two or more PFB files. We also introduce an open source software development kit (SDK) called PyPFB for creating, exploring and modifying PFB files. We describe experimental studies showing the performance improvements when importing and exporting bulk biomedical data in the PFB format versus using JSON and SQL formats.

## Author summary

Many biomedical data sets have a unique structure that encapsulates and describes the data. When working with these datasets it can be difficult to keep track of the overall structure and ontologies that define the individual properties. PFB was developed so that working with this type of data is made simpler. This allows anyone interacting with the data to bring this fully self-describing dataset in one file anywhere and do analysis over the phenotypic and biological data contained within it. PFB was devleoped over the Avro serialized data format which helps researchers and commons operators to make data schema updates as well as change references to external ontolgies. In this work we show the advantages to using PFB as a bioinformatic tool and how it is used to enable fast sharing of large biomedical research data sets. The results also show that PFB is bringing significant speedups for storing and sharing structured biomedical datasets.

This is a *PLOS Computational Biology* Software paper.

## 1 Introduction

We introduce a self-describing format based upon Avro [1] for bulk structured biomedical data called the Portable Format for Bioinformatics (PFB) that encapsulates a data model, a data dictionary, the data itself, and pointers to third party controlled vocabularies. The data types supported include persistent identifiers to data objects stored in public and private clouds and manifests (also known as bundles or containers) of these.

The GA4GH Data Repository Service (DRS) [2] is emerging as a standard for *data objects* stored in public clouds to support cloud based biomedical platforms, but there is no analogous format for the long term storage of bulk *structured data* in public clouds, such as clinical or biospecimen data. By “bulk structured data,” we mean data that is stored in a database, used for operational purposes, and exported in a bulk format for importing into another system, for archival purposes, or for some other purpose. It is common for a cloud-based data platform to manage data with both a database and with data objects, where there are DRS references to the data objects managed by the database [3]. Importantly, unlike data objects, structured data requires a data schema, and over time, it is unfortunately common for the data and data schema to become separated if they are stored in separate files.

Examples of structured data include: 1) EHR data; 2) real world evidence (RWE) data; 3) clinical trial data; 4) pre-clinical data / experimental data. Each of these different types of data have common standards, including FHIR [4] and OMOP [5] for EHR and RWE; CDISC [6] for clinical trials; and discipline specific standards for pre-clinical and experimental data.

When considering formats for bulk structured data, it is sometimes helpful to distinguish between two types of systems and the bulk data formats they typically use (see, for example, [7, Chapter 4]). The first type are systems supporting health care and medical research and the second type are systems supporting patient encounters with health care providers. The first type of system include data repositories and data registries that are patient-centered and purpose driven. An example of a purpose in this context is outcomes research. The second type of system include electronic health records (EHR) systems that are visit focused and transactional. For simplicity, we will use the terms *research systems* and *operational systems* respectively for these two types of systems.

Operational systems are well supported by the FHIR standard [4], but there is not yet the same level of consensus for accessing bulk data for research systems. As an example of a research system that benefit from data formats such as PFB are data ecosystems consisting of multiple interoperating data platforms, data repositories, knowledgebases and other components [3, 8] in which bulk structured data must be exported and imported between data ecosystem components. As another example, patient-centered bulk data are usually the preferred format for machine learning and AI research, and, in this context, are referred to as AI-ready datasets. Although PFB can be used more generally for any type of structured scientific data, for simplicity we only consider biomedical data in this paper.

The importance of data serialization formats for structured data was broadly recognized with Google’s public introduction of Protocol Buffers in 2008 [9]. Protocol Buffers were used internally within Google prior to that. As stated succinctly in [9], a data serialization formats is a “flexible, efficient, automated mechanism for serializing structured data—think XML, but smaller, faster, and simpler.” There are a variety of serialization formats available, including Apache’s Thrift, developed by Facebook, and Apache Avro, which is used within the Hadoop Project.

We follow [9] in explaining the main reasons for adapting serialization formats for working efficiently with structured data in data commons. Serialization formats are:

- Extensible: New fields can be added to a serialization format in a forward-compatible way.
- Efficient: Data is serialized into a compact binary representation for writing, reading and transmission, which can sometimes improve performance by a factor of 10x or more.
- Portable: Serialization formats allow different applications to exchange data simply by importing and exporting.
- Type safe: Programming errors resulting from incorrect types can be quite difficult and labor intensive to track down. Serialization formats enforce correct types.

The alternative is often to use custom code with long chains of “if statements” checking for different versions of the clinical data.

**Fig 1:**
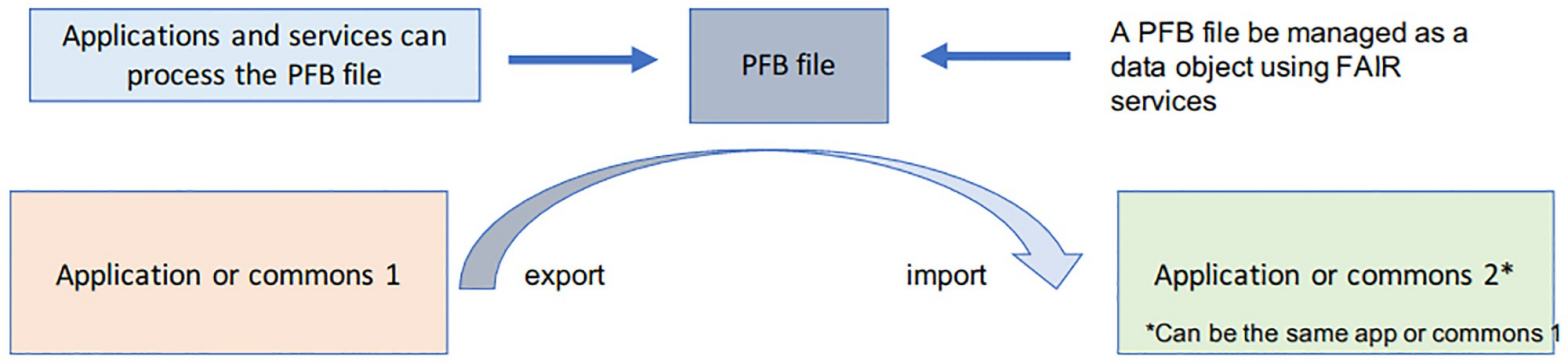
A data commons or application in a data ecosystem can export a PFB file and the same or another data commons or application can import it.

We are specifically interested in an application independent and system independent serialization format for importing and exporting: 1) the data schema and other metadata associated with structured data, 2) pointers to third party controlled vocabularies and standards, and 3) the data itself. See Fig 1.

PFB supports storing, editing, and versioning of bulk structured biomedical data, such as clinical, phenotype, or biospecimen data. PFB inherits the advantages of Avro and is fast and extensible, which allows for quick imports and exports within and between systems. Using PFB, it is straightforward to save snapshots of the data schema and associated data for versioning and archival purposes. In particular, PFB files can be assigned persistent digital identifiers and exposed through APIs to make bulk clinical data packaged as a PFB file findable, accessible, interoperable and reusable (FAIR) [10]. PFB also allows biomedical data to be exported, processed using any technologies desired, and then re-imported.

PFB is designed to be integrated into a GA4GH compliant environment as a GA4GH evolves. Today, in systems such as Gen3, i) a PFB file is assigned a GA4GH DRS identifier; ii) metadata is associated with the DRS identifier that describes the PFB file; and, iii) data objects referred to within the PFB file use DRS identifiers. As GA4GH establishes a standard mechanism to assign metadata to a DRS identifier, this practice can be adopted by systems such as Gen3 that use PFB.

We conclude this section by describing some of the existing formats for both general purpose scientific data and biomedical data. The most common way that clinical data is managed is in a database and therefore the most common way that a system imports and exports clinical data is by importing and exporting a collection of database tables in a TSV or CSV format. Although not widely used in the biomedical community, the W3C standard CSV on the Web supports CSV formats, but is not designed for bulk data, as is the focus of PFB.

There are quite a few attempts to create various data interchange formats for clinical data, and, more generally, biomedical data. These include the DataMed DATS format [11], which is a JSON format targeted at making data discoverable. The Fast Healthcare Interoperability Resources (FHIR) is a standard describing data formats and elements and an application programming interface for exchanging electronic health records. The standard was created by the Health Level Seven (HL-7) International health-care standards organization [12]. FHIR is built over JSON, XML and RDF.

GA4GH Phenopackets defines a schema that is a phenotypic description of a patient/sample in the context of rare/common diseases or cancer [13]. The schema and data is also serialized, in this case with Protocol Buffers. We described in Section 2.1 some of the differences between Avro and Protocol Buffers and why we chose Avro for PFB. Also, as mentioned, PFB can be used with any data model, while Phenopackets are particularly focused on phenotypic data.

Another widely used format for scientific data, especially for geospatial data and images is the Hierarchical Data Format (HDF) [14]. In the context here, HDF files, and in particular, HDF files representing images would be assigned a DRS identifier, which would be referenced by the PFB file, similar to how BAM files would be managed. This is for example, common with single cell transcriptomics images.

RO-Crate is a lightweight approach for packaging research data and the associated metadata [15]. RO-Crate is based on Schema.org annotations in JSON-LD. An RO-Crate object is a structured archive associated with a research project that includes identifiers, provenance, relations and annotations, and hence is targeted at capturing more information than a PFB object.

## 2 Design and implementation

### 2.1 Avro PFB format

Avro is an Apache data serialization format that is extensible, efficient, and portable (https://avro.apache.org/). Avro is a flexible serialization format designed to support arbitrary data. Using Avro as the base for PFB, enables PFB to take advantage of the numerous benefits from Avro, such as schema evolution and the large amount of tooling developed to work with Avro.

We chose Avro over other common serialization formats because it has the following advantages. Avro is self-describing, so Avro stores both the data and schema in one file that results in easier sharing and storing of the resulting files. The Avro schema is dynamic so there is no need for recompilation of programs to support new schemas. Avro is converted between a computationally efficient binary format, and a human readable JSON format. The ability to convert between the two formats provides the ability to cover a large variety of use cases for PFB. See Table 1 for a summary of some of the reasons that Avro was chosen over Protobuf and other data serialization formats for PFB.

**Table 1:** A comparison of Avro versus Protobuf for managing biomedical data.

	Avro	Protobuf
self-describing	Y	N
schema-evolution	Y	Y
dynamic schema	Y	partially, needs recompiling
compilation required	N	Y
Hadoop support	Y	requires 3rd party library
JSON schema	Y	requires separate IDL for schema

**Fig 2:**
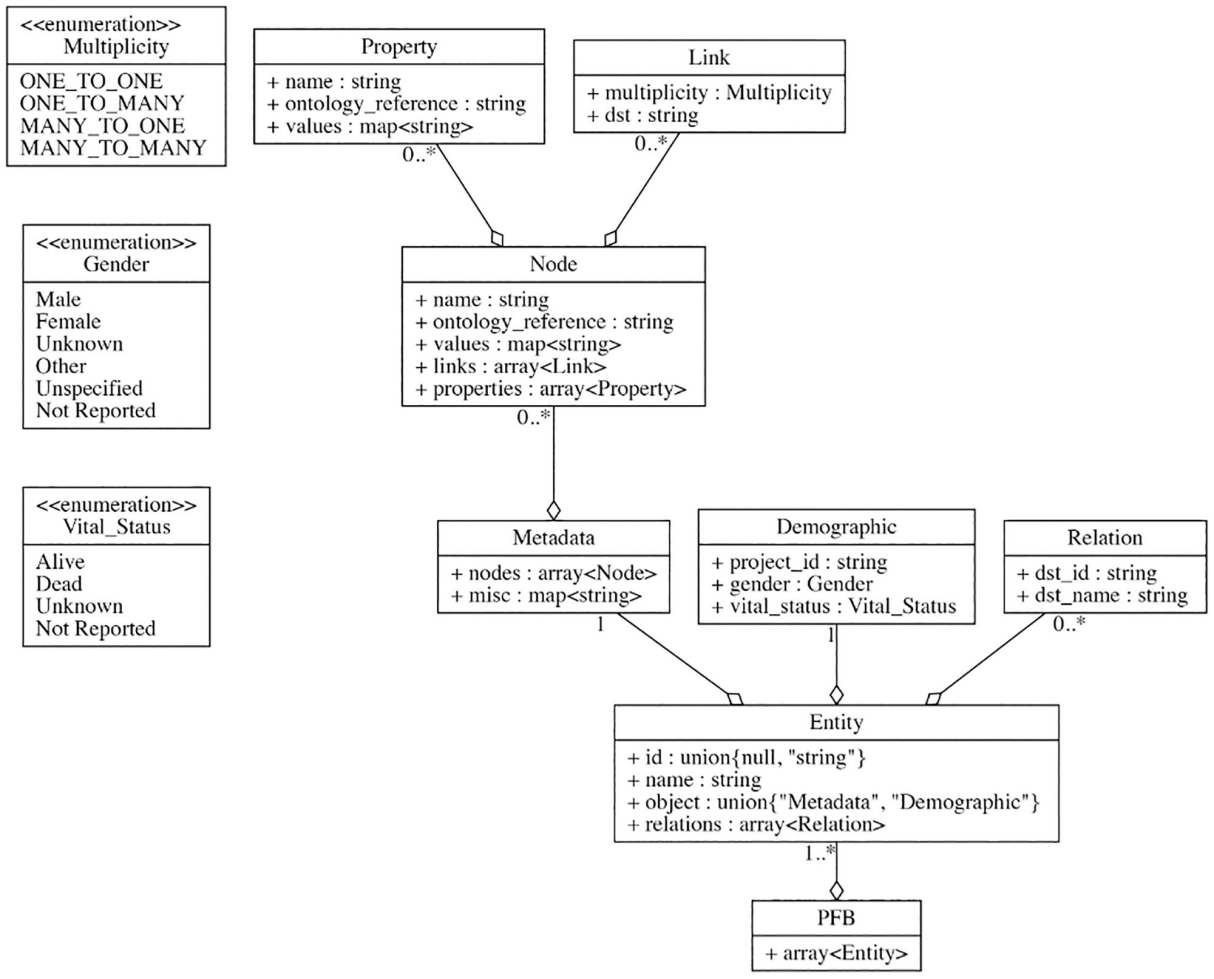
The graphical representation of PFB schema for a data model with only a Demographic node.

Although, PFB can be defined for a variety of different types of data models, in this paper we focus on graphical data models, since this is the type of data model used by Gen3 data commons [3], which are are used for the experimental studies reported in this paper. In a Gen3 commons, a data dictionary is defined, as well as a graph based data model specifying relationships between different data elements. For simplicity, we refer to the data dictionary and the graph based data model collectively as the *data dictionary*. The data dictionary define the nodes and edges on the graph data model as well as the properties for each node. Each node has a type, such as number, string, enumeration, or date. The valid ranges for the properties are included in the data dictionary definitions. In general, each node in the data dictionary also includes a reference to an appropriate third party controlled vocabulary, ontology, or other standard. Popular ontologies that are referenced in PFB files include the NCI Thesaurus [16], SNOMED CT [17], Disease Ontology [18], and Human Phenotype Ontology [19]. Fig 2 shows a graphical representation of the schema as it is encoded in PFB for a simple case where there is only one node present, Demographic.

### 2.2 Encoding a graphical data model in PFB

A formal definition of PFB can be found https://uc-cdis.github.io/pypfb/.

To further understand PFB it is useful to look at the Avro IDL format [1] for PFB and how a single node of a graphhical data model is encoded in PFB.


record Node {
    string name;
    string ontology_reference;
    array<Link> links;
    array<Property> properties;
}


Each node is given an identifier, referred to in PFB as the name. This identifier is unique to a single node in the PFB file. The ontology reference property is defined as a string type called ontology_reference. The value of this string is the URI to the appropriate ontology for the node. The properties and links are both arrays allowing an arbitrary number of either to be present on a single node.

The properties for each node are defined as follows:


record Property {
    string name;
    string ontology_reference;
    map<string> values;
}


Each property is given an identifier (name), which is unique to the node in which it is attached. The ontology reference is again the URI to the appropriate ontology for this property. The values are a map data structure, with keys being strings (Avro specification supports only strings for keys) and types being strings. The map contains the ontology_reference as a key so that each value can also be referenced to a 3rd party ontology. The map can also be used to store the source information, or comments about the value.

The edges in the graphical data model are specified on each node as a link pointing to the parent node. The links themselves are described as follows in the Avro IDL:


enum Multiplicity {
    ONE_TO_ONE,
    ONE_TO_MANY,
    MANY_TO_ONE,
    MANY_TO_MANY
}

record Relation {
    string dst_id;
    string dst_name;
}

record Link {
    Multiplicity multiplicity;
    string dst;
}


As can be seen, the link contains a string pointing to the name of the destination nodes. Each link also contains a multiplicity property describing if the link allows only one-to-one, one-to-many, many-to-one, or many-to-many relations with the parent nodes.

For clarity, we note that the nodes and relationships coded in PFB correspond to a graphical data model that describes the structured data. As noted above, PFB formats can be defined for data models other than graph data models. PFB is also silent on what the graph data model encodes. In particular, although PFB could be used to encapsulate a knowledge graph, as an instance of a graph data model, there is no specific support in PFB for knowledge graphs beyond the ability to encapsulate data and a data model, with the data model containing optional links to third party controlled vocabularies or ontologies.

To ease the storing and processing of a PFB, PFB uses a wrapper type called Entity. An Entity helps to store the data and metadata (id, node name inside field name and Relation to other nodes in relations) for this record separately. Each Relation stores the destination node name and destination record id.

The Metadata type stores ontology references for both nodes and their properties. To properly link each node with its properties to ontology references, PFB stores the name of the ontology references and additional properties inside values. Values can include all the information required for an ontology reference, e.g., URL, ontology version, ontology name. It also stores list of properties for a node and its ontology references in the same format.

The Avro schema for PFB is generated from the data dictionary. Each PFB file stores a list of records of type Entity.

## 3 Results

### 3.1 PFB SDK

We have developed an open source Python-based SDK for working with PFB files called PyPFB that is available from Github (https://github.com/uc-cdis/pypfb). PyPFB allows users to create, explore, modify, import and export PFB files. PyPFB is licensed under Apache License 2.0. The experimental studies below were done with PyPFB.

As an example, with PyPFB, a user can create a stub PFB file from a data dictionary and then populate it with data from JSON files. By a stub PFB file, we mean a PFB file without data. PyPFB can also be used to import and export PFB from a system. PyPFB commands include:


From
usage: pfb from [OPTIONS] COMMAND [ARGS]…
Generate PFB from other data formats.

To
Usage: pfb to [OPTIONS] COMMAND [ARGS]…
Convert PFB into other data formats.

Show
Usage: pfb show [OPTIONS] COMMAND [ARGS]…
Show records of the PFB file.
Specify a sub-command to show other information.

Make
Usage: pfb make [OPTIONS] NAME
Make a blank record according to given NODE schema in the PFB file.

Rename
Usage: pfb rename [OPTIONS] COMMAND [ARGS]…
Rename different parts of schema.

Importer
Usage: pfb importer [OPTIONS] COMMAND [ARGS]…
Create job to import PFB to commons.


### 3.2 Experimental setup

The experimental studies were performed using Gen3 data commons running in AWS and using the PyPFB for exporting and importing PFB files. S3 was used to store the data objects, including BAM, FASTQ, and imaging files. The data commons used for these studies included over 5 PB of data in S3 buckets.

The clinical and other structured data in the Gen3 commons were stored in a PostgreSQL database running in AWS. The structured data was extracted, transformed and loaded into Elasticsearch to support queries. PostgreSQL uses a db.r4.large AWS instance, while Elasticsearch uses a m4.large.elasticsearch instance.

Gen3 uses microservices for authentication, authorization, indexing, querying, and accessing the object data and structured data. The experimental studies used 5 AWS instances of EC2 services at a t3.xlarge size for managing the microservices, except for the indexing microservice which used a db.r4.large instance.

For the tests importing and exporting PFB described in this section, we used simulated data generated by an open source data simulator made specifically to simulate structured data for Gen3 data commons (https://github.com/uc-cdis/data-simulator/). This tool simulates data based upon a Gen3 data dictionary. The tool verifies the dictionary, builds an appropriate graph structure for a Gen3 data model, and populates JSON files with simulated data.

For this series of experiments, we used synthetic data based on a graph data model with 26 nodes and 399 attributes. The choice of 26 nodes and 399 attributes was arbitrary but corresponds approximately to the number of nodes and attributes arising in the Gen3 data commons mentioned above. We have not seen any change in import/export performance as the number of attributes ranges between 100 and 10,000 beyond that associated with the total data size, which is captured by increasing the number of records.

Comparing read and write speeds of PFB versus JSON was done using a Macbook Pro 13-inch, 2017 with Intel Core i5-7267U CPU @ 3.10GHz, 16 GB 2133 MHz LPDDR3 and 500 GB Flash Storage.

### 3.3 Transforming PFB files

As a simple example of how PFB can be used, if data elements in a data platform refer to the CDISC standard (https://www.cdisc.org/), the data could be exported to a PFB file, the CDISC references could be replaced with references to the NCI Thesaurus (NCIt) (https://ncit.nci.nih.gov/ncitbrowser/) by processing the PFB file, and the new transformed PFB file with references to NCIt could be re-imported into the data platform. The supporting information contain a Python notebook that illustrate this.

### 3.4 Importing and exporting PFB files

In the first set of experiments, we imported data into a Gen3 data commons using Sheepdog, which is the Gen3 data submission system, versus bulk loading the data using PFB. For this series of experiments, we generated simulated data in JSON format corresponding to a graph data model with 26 nodes and 399 total attributes as described above. We then used PyPFB to convert the JSON data into PFB. We tested submissions from 10 records per node(260 records in total) to 100000 records per node (2.6 million records in total) to compare the submission times of the current Gen3 data submission system, compared to importing the same data using PFB. We ran these tests 3 times and the shown results are the averages. The number of attributes is the number of fields submitted per record. The results are in Table 2.

**Table 2:** Import time comparison between Gen3 Import and PFB. * denotes that the submission did not complete and would take over 40 hours.

Records	Attributes	Size	JSON Import (sec)	PFB Import (sec)	Improvement
260	399	256K	36.55	50.74	0.72
2600	399	1.9M	244.60	53.34	4.58
26000	399	19M	2302.65	93.58	24.60
260000	399	184M	19596.05	462.07	42.40
2600000	399	1.9G	*	4134.68	*

As we can see from Table 2, the PFB import is over an order of magnitude faster than the current native Gen3 data import system. We then tested the export time between a dump to PFB file and a SQL dump through Gen3’s native export. This was done using the same simulated data. See Table 3.

**Table 3:** Export time comparison between PFB and Gen3.

Records	Attributes	Size	JSON Export (sec)	PFB Export (sec)	Improvement
260	399	256K	2	3.2	0.6225
2600	399	1.9M	2	5.5	0.363
26000	399	19M	11.6	11	1.054
260000	399	184M	87.5	69.7	1.255
2600000	399	1.9G	912.5	551	1.656

Next, we look at the size of the bulk data files after compression. For JSON compression, we used *tar.bz2*, and, for PFB compression, we used Avro’s built in compression codec.

Table 4 shows the sizes of compressed JSON to PFB. For Avro compression, we used the default Avro support for compression (the deflate codec). Avro allows us to submit a compressed PFB file with little to no overhead for compression.

**Table 4:** Size comparison between JSON and PFB, [c] denotes the data was compressed.

Records	Attributes	JSON	PFB	PFB [c]
260	399	256K	187K	108K
2600	399	1.9M	867K	347K
26000	399	19M	7.5M	3.0M
260000	399	184M	74M	29M
2600000	399	1.9G	542M	239M

We also performed several experiments using the structured project data from three large scale cloud-based data platforms: the KidsFirst Data Resource [20], the NHLBI BioData Catalyst system [21], and the NCI Genomic Data Commons (GDC) [22]. We took: 1) PostgreSQL dumps and 2) PFB exports from each system. A summary is in Table 5. From Table 5, we can see that database exports to PFB can be much smaller than SQL.

**Table 5:** Size comparison of existing commons between SQL dump and PFB dump.

Data Commons	# records	# attributes	SQL	PFB
GDC	5740427	795	30.7GB	3.6GB
KidsFirst	153431	384	277MB	39MB
BioData Catalyst	3024605	739	33GB	981MB

In a final set of experiments, we compared the raw speed of reading PFB and JSON to and from disk. The results are shown in Table 6. Note that, as expected, reading clinical data using PFB versus JSON is about 4.3 times faster, while writing PFB versus JSON is about 2.6 times faster. Note that the other experiments above were concerned with the relative speed of importing and exporting structured data into and out of a Gen3 data commons using the Gen3 native services versus PFB services.

**Table 6:** Read/write speed comparison between PFB and JSON from/to disk.

Format	Records	Size (GB)	Read speed (records/s)	Write speed (records/s)
PFB	2600000	0.568	6.5 × 10^6^	1.8 × 10^6^
JSON	2600000	1.921	1.5 × 10^6^	0.7 × 10^6^

## Availability and future directions

A common use of PFB is to make bulk clinical data FAIR. The structured data in a data platform can be exported to a PFB file, a digital ID can be assigned to the PFB file, and the PFB file can then be uploaded as a data object to a data lake, data commons, or other data repository.

Another common use is to version bulk clinical data by taking a snapshot of the data and exporting a PFB file, which can be managed as a data object with a DRS identifier. Clinical data can be rolled back to a previous version by importing the associated PFB file.

Submitting data to a production data platform can be challenging and labor intensive. One of the advantages of using PFB and related formats is that a separate system can be used to curate and prepare data for submission. The data can then be checked for compliance with all the data submission requirements and then exported as a PFB file. The data can then be bulk uploaded to a production data platform.

All code related to the SDK is available at https://github.com/uc-cdis/pypfb. All data used for testing, as well as showcasing uses of PFB in the paper is availiable at https://github.com/uc-cdis/pfb-paper-artifacts

## Conclusion

Over the past several years, several large scale cloud-based data commons and data clouds have been developed, including the NCI Genomic Data Commons, the Kids First Data Resource, and the NCI Cloud Resources [3]. These systems manage petabytes of data and make use of cloud-based bioinformatics workflows that require hundreds of thousands to millions of core hours. These systems use a data lake architecture with digital object IDs to manage large genomic and imaging files, which makes this data findable, accessible, interoperable and reusuable (FAIR). To date, there has not been a FAIR approach that has proved effective for managing the clinical and other structured data that these data clouds and data commons contain and can still be efficiently be processed in bulk by third party applications and services.

We introduced the Portable Format for Biomedical data (PFB) for this purpose, developed a SDK for it, integrated the SDK with a Gen3 data commons, and provided several experimental studies showing that PFB can manage the structured biomedical data that current data clouds and data commons contain. In particular, we showed that this approach can significantly speed up the importing and exporting of bulk clinical data and, in this way, improve the interoperability of data ecosystems consisting of multiple data commons, data repositories, and other data platforms. Data ecosystems often include data from a wide variety of different disciplines, and a general format like PFB has advantages compared to discipline specific data formats, such as FHIR.
